# Telehealth as a Substitute for a Usual Source of Care for Prescription Medications

**DOI:** 10.5195/ijt.2024.6672

**Published:** 2025-01-15

**Authors:** David Shilane, Ashwathi Nair

**Affiliations:** Program in Applied Analytics, School of Professional Studies, Columbia University, New York, New York, US

**Keywords:** Chronic disease management, Primary care, Telehealth, Usual source of care, Prescription medications, Rural health

## Abstract

This study investigates how effectively telehealth utilization (THU) can substitute for a usual source of care (USC) for taking prescription medication using data from the 2020–2022 National Health Interview Survey. We analyzed data for 69,581 patients. Of these, 5,994 patients (8.6%) lacked a USC. THU was 37.3% for patients with a USC and 15.8% for those without. For patients with no USC or THU, 25.4% had taken a prescription medication within 12 months, while patients with THU but no USC had a rate of 75.4%. In essentially all subgroups, telehealth was associated with substantially higher rates of taking prescription medications. Multivariate logistic regression showed that THU was associated with a 7.39-fold increase (95% CI: 6.19–8.84) in the odds of taking a prescription medication.

A usual source of care (USC) is a healthcare facility that a patient typically visits for primary care appointments (Kim et al., 2012). A USC can refer to a specific primary care provider (PCP) such as a doctor or nurse practitioner, a specific clinic, or a specific PCP within a specific clinic (Kim et al., 2017). With a USC, patients benefit from improved quality and timely access to care (Kim et al., 2017). A USC facilitates ongoing relationships with PCPs, the familiarity of providers with the patient's health and medical history, and connections to specialists within the PCP's network or the facility's network (DeVoe et al., 2003). Continuity of care is an important indicator of quality and contributes positively to the patient's experience (Tarrant et al., 2014). Relative to a usual standard of primary care, instituting comprehensive primary care services in Germany was associated with a 9.2% reduction in all-cause hospitalization for elderly patients, 5.1% reduction for all-cause hospitalization for patients with diabetes, and a 7.5% reduction in cardiovascular hospitalizations for patients with coronary heart disease (CHD) (Sawicki et al., 2021). For patients hospitalized with an acute myocardial infarction, lacking a USC was associated with a 92% increase in the hazard of mortality within 1 year compared to having a strong relationship with a USC (Spatz et al., 2014). Having a usual source of care is also associated with improved utilization of a range of preventive screening services (DeVoe et al., 2003, Kim et al., 2012), with smaller gaps based on race or ethnicity for patients with a USC than those without a USC (Corbie-Smith et al., 2002). Having a USC can promote more regular appointments and follow-up (Xu, 2002). In a range of preventative services, having a USC is comparable in quality to having a primary care provider (Xu, 2002). Relative to having a USC without a primary care provider (PCP), having both a PCP and a USC was associated with 23% reductions in hospital admissions and 29% reductions in emergency department (ED) visits (Kim et al., 2017). Over the last few decades, patients have shifted from PCPs (43% reduction in “person USC”), with an 18% increase in facility USCs and a 10% increase in patients with no USC or PCP (Liaw et al., 2017).

However, approximately 20.8% of patients in the United States lack a USC (DeVoe et al., 2003). Patients without a USC may have difficulty securing appointments when needed (Xu, 2002). The management of chronic diseases is greatly impacted by a lack of access to primary care (Kim et al., 2012). These challenges can create obstacles to obtaining prescription medications when a patient lacks a USC (Spatz et al., 2014). Within the United States, reduced availability of primary care providers has been associated with increased rates of mortality (Engström et al., 2001).

Relative to patients living in urban or suburban areas, patients living in rural areas of the United States were 21.9% less likely to have a USC (DeVoe et al., 2003), even while facing a greater burden of disease (Baernholdt et al., 2012). Compared to suburban or urban areas, the supply of providers is 38% lower for primary care and 70% lower for specialists in rural areas on a per-capita basis (Win, 2015). Rural patients are comparatively older, with a greater degree of medical needs and higher propensity for social isolation (Baernholdt et al., 2012). For behavioral health conditions, there is both an increased prevalence in rural areas and a shortage of clinical providers (Lutfiyya et al., 2012). Rural patients typically have to travel longer distances for medical care, with an average of 96 kilometers required to visit specialists (Win, 2015). Among patients who survived cancer and were at least 65 years old, patients living in rural areas opted to delay or forego care due to cost at 66% higher rates relative to patients living in non-rural areas (Palmer et al., 2013). Reduced utilization of primary care for patients of low socioeconomic status can increase the existing disparities in health outcomes and is associated with greater rates of preventable hospitalization (Kangovi et al., 2013).

Telehealth utilizes technology to allow patients and healthcare providers to conduct appointments remotely, either by phone or video conference (Silver et al., 2021). Telehealth has the potential to serve as a substitute for or supplement of primary care (Baltaro et al., 2023, Pidgeon, 2015). Remote appointments offer greater convenience, which can reduce obstacles to care driven by commuting concerns (Pidgeon, 2015), geographic barriers (Ferrer-Roca et al., 2010), or mobility issues (Westby et al., 2021). Telehealth appointments can offer patient access to a wider network of providers (Dixit et al., 2021). Telehealth appointments can allow a patient to meet with a provider and fill a prescription in a timelier manner (Baltaro et al., 2023). Telehealth interventions have shown improvements in medication adherence (Fuentes et al., 2022), especially in rural areas (Baldoni et al., 2019). During the first year of the COVID-19 pandemic, telehealth was associated with increases in the medication possession ratio of 3.4% for patients with diabetes and 3.1% for patients with hypertension (Cho et al., 2024). Compared to in-person specialty care, telehealth visits with a specialist can lead to similar results in terms of quality of life and health status (Ferrer-Roca et al., 2010). Telehealth has also shown associations with reductions in ED visits, hospital admissions, and length of stay in the hospital for patients with a range of chronic medical conditions (Bashshur et al., 2014).

This study investigated the question of how effectively telehealth can facilitate utilization of prescription medications among patients without a USC. The primary aim was to quantify and compare the percentage of patients without a USC who take prescription medications with and without telehealth utilization. We also compared these groups to patients who have a USC. These analyses included comparisons of subgroups based upon demographic factors, health conditions, behavioral health conditions, challenges of ability, and poverty status.

## Data and Variables

We gathered publicly available, de-identified, patient-level data for patients at least 18 years old in the United States from the National Health Interview Survey (NHIS) in the years 2020 through 2022 (National Center for Health Statistics, 2021–2023). The NHIS data provide a large, nationally representative sample of patient health and healthcare utilization within the United States. The NHIS added a question about telehealth utilization (THU) starting in the middle of 2020 in response to the emerging COVID-19 pandemic.

In response to the survey, the patients provided self-reported information on their usage of a USC, telehealth, filling prescriptions, and their health profile. We created a binary variable for a usual care facility based upon affirmative responses (a usual care facility or more than one such location) versus negative responses (no such facility). The survey assessed THU as a binary variable based on whether the patient had held a remote appointment by video or phone within the past 12 months. Utilization of prescriptions was also based on a binary response of whether the patient had taken a prescription medication at any point in the past 12 months.

The NHIS also includes data on a range of chronic conditions, behavioral health conditions, challenges of ability, demographic variables, and income as a ratio to the poverty threshold, among other variables. The chronic conditions included binary indicators of asthma, coronary artery disease (CHD), chronic obstructive pulmonary disease (COPD), cancer, diabetes, hypertension, along with a binary indicator of at least one of these conditions. The behavioral health conditions included binary indicators of anxiety and depression. The challenges of ability consisted of binary indicators of visual impairment, hearing impairment, difficulties with communication, running errands, using the hands or fingers, raising arms, and self-care, along with a binary indicator of at least one of these challenges. The demographic variables included age groups, gender groups, and rural geography versus suburban/urban geography. Income groups were included as a ratio of the poverty threshold.

## Methods

We categorized the patients based on USC and THU. Within each group, we calculated and compared the percentage of patients who had utilized prescription medications within the past 12 months. These rates were also calculated within subgroups based upon calendar year, demographic variables, chronic conditions, behavioral health conditions, and income groups as a ratio to the poverty threshold.

Logistic regression was used to assess the differences in the percentage of patients who took prescription medications between groups based on USC and THU while adjusting for the measured variables, which included demographics, survey year, chronic conditions, behavioral health conditions, challenges of ability, education, income group, and food barriers. Then, restricting attention to patients without a USC, we statistically compared the characteristics of the patients who utilized telehealth to those who had not.

## Results

The data included records for 69,581 patients who were at least 18 years old. Table 1 displays the sample size by USC status and THU. Overall, 5994 patients (8.6%) did not have a USC. Among these patients, 15.8% utilized telehealth. For patients with a USC, the rate of THU was 37.3%.

**Table 1:** Sample Size by USC Status and THU

	No THU	THU	% THU
**No USC**	5,044	950	15.8%
**Has a USC**	39,844	23,743	37.3%
**% with No USC**	11.2%	3.8%	

Table 2 shows the percentage of patients who utilized telehealth by year and USC status. In 2020, 14.3% of the patients without a USC had THU, while this figure was 38.0% for patients with a USC. In 2021, the rates rose modestly in both categories, with THU rates of 16.1% for patients without a USC and 41.1% for patients with a USC. In 2022, 16.3% of patients without a USC utilized telehealth, but the rate for patients with a USC fell to 32.8%. These utilization figures reflect different periods of the COVID-19 pandemic, with the peak of case rates in 2020.

**Table 2:** The Rates of Telehealth Utilization (THU) by Year and USC Status

Year	% THU Patients with no USC	% THU Patients with a USC
**2020**	14.3%	38.0%
**2021**	16.1%	41.1%
**2022**	16.3%	32.8%

Table 3 shows the overall rates of telehealth utilization by USC and THU. A total of 25.4% of patients with no USC or THU had taken a prescription medication, while these rates were 75.4% (THU without USC), 65.7% (USC without THU), and 88.9% (USC and THU) in the other categories.

**Table 3:** Percentage of Patients Who Took a Prescription Medication in the Past 12 Months Based on USC Status and THU

	No THU	THU
**No USC**	25.4%	75.4%
**Has a USC**	65.7%	88.9%

Table 4 summarizes the rates of taking prescription medications by USC and THU status for patients with a range of chronic conditions. Aggregating to any patient with at least one of these conditions, 39.8% of patients without a USC or THU had taken a prescription medication, while these rates were 86.9% (THU without USC), 86.0% (USC without THU), and 95.8% (USC and THU) in the other categories. For each condition, the percentages taking a prescription medication were at least 20% higher for patients with THU among those without USC, and in some cases (asthma, COPD, cancer), the rates effectively doubled.

**Table 4:** Percentage of Patients with Chronic Conditions Who Took a Prescription Medication in the Past 12 Months Based on USC Status and THU

Condition	USC: No THU: No	USC: No THU: Yes	USC: Yes THU: No	USC: Yes THU: Yes
**1+ Chronic Condition**	39.8% N = 1,290	86.9% N = 411	86.0% N = 20,377	95.8% N = 15,040
**Asthma**	33.5% N = 543	84.3% N = 191	74.8% N = 4,602	93.7% N = 4,299
**CHD**	62.3% N = 61	95.7% N = 23	95.7% N = 2,163	98.9% N = 2,005
**COPD**	45.8% N = 96	94.3% N = 35	91.6% N = 1,906	98.4% N = 1,887
**Cancer**	42.0% N = 162	84.1% N = 63	87.2% N = 4,720	95.8% N = 3,953
**Diabetes**	72.7% N = 88	95.7% N = 47	96.2% N = 3,741	98.8% N = 3,466
**Hypertension**	48.0% N = 638	91.9% N = 198	91.7% N = 14,284	97.7% N = 10,333

Table 5 shows the percentage of those who took prescription medications by USC and THU categories for patients with behavioral health conditions. For patients with anxiety, 46.7% of patients without USC or THU had taken a prescription medication, while these rates were 88.6% (THU without USC), 84.7% (USC without THU), and 96.0% (USC and THU) in the other categories. Likewise, for patients with depression, these percentages were 45.4% (no USC or THU), 87.9% (THU without USC), 84.6% (USC without THU), and 96.1% (USC and THU).

**Table 5:** Percentage of Patients with Behavioral Health Conditions Who Took a Prescription Medication in the Past 12 Months Based on USC Status and THU

Condition	USC: No THU: No	USC: No THU: Yes	USC: Yes THU: No	USC: Yes THU: Yes
**Anxiety**	46.7% N = 424	88.6% N = 298	84.7% N = 4,705	96.0% N = 6,209
**Depression**	45.4% N = 500	87.9% N = 314	84.6% N = 5,361	96.1% N = 6,659

Table 6 provides information on the percentage of patients with challenges of ability who took prescription medications by USC and THU status. Aggregating to at least one condition, these rates were 32.8% (no USC or THU), 83.6% (THU without USC), 78.6% (USC without THU), and 94.4% (USC and THU). Among patients without a USC, the percentage taking prescription medications was at least 66% higher in all categories and was at least 100% higher for most subgroups.

**Table 6:** Percentage of Patients with Challenges of Ability Who Took a Prescription Medication in the Past 12 months Based on USC Status and THU

Challenge of Ability	USC: No THU: No	USC: No THU: Yes	USC: Yes THU: No	USC: Yes THU: Yes
**1+ Challenge of Ability**	32.8% N = 1,369	83.6% N = 323	78.6% N = 14,067	94.4% N = 10,441
**Communication**	33.2% N = 205	93.8% N = 48	77.6% N = 1,815	95.2% N = 1,314
**Running Errands**	48.0% N = 171	91.2% N = 80	87.3% N = 2,793	97.5% N = 2,964
**Hands/Fingers**	44.1% N = 161	89.7% N = 58	89.1% N = 3,216	97.6% N = 3,192
**Hearing Impairment**	36.3% N = 509	86.5% N = 126	81.7% N = 6,458	94.8% N = 4,642
**Raising Arms**	50.7% N = 69	94.4% N = 36	90.3% N = 1,590	97.4% N = 1,679
**Self-Care**	54.9% N = 71	91.1% N = 45	89.5% N = 1,309	97.8% N = 1,556
**Visual Impairment**	30.3% N = 766	84.7% N = 189	76.0% N = 6,645	94.1% N = 4,972

Table 7 shows the percentage of patients who took prescription medications by USC and THU categories among subgroups of age, gender, and geography. Older patients showed higher rates of taking prescription medications. Male patients had markedly lower percentages than other patients, while patients in rural areas and suburban/urban areas had similar percentages of prescription medication usage. In all these categories, the percentage of patients without a USC who took prescription medications was approximately 2–3 times higher for patients with THU than without.

**Table 7:** Percentage of Patients by Age Group, Gender, and Geographic Region Who Took a Prescription Medication in the Past 12 Months Based on USC status and THU

Demographic Subgroup	USC: No THU: No	USC: No THU: Yes	USC: Yes THU: No	USC: Yes THU: Yes
**Age: 18–34**	23.2% N = 2,157	72.2% N = 461	40.2% N = 7,660	78.3% N = 3,909
**Age: 35–49**	21.3% N = 1,493	72.0% N = 250	50.1% N = 8,734	82.7% N = 5,273
**Age: 50–64**	29.6% N = 950	81.3% N = 139	70.1% N = 10,332	91.4% N = 6,400
**Age: 65–74**	37.2% N = 312	87.5% N = 72	84.7% N = 7,312	95.5% N = 4,817
**Age: 75+**	48.5% N = 132	96.4% N = 28	90.9% N = 5,806	96.6% N = 3,344
**Gender: Not Male**	34.3% N = 1,820	81.4% N = 501	70.2% N = 21,176	89.5% N = 14,375
**Gender: Male**	20.3% N = 3,224	68.6% N = 449	60.6% N = 18,668	87.9% N = 9,368
**Geography: Urban/Suburban**	25.2% N = 4,403	75.5% N = 882	63.9% N = 32,626	88.3% N = 21,035
**Geography: Rural**	26.4% N = 641	73.5% N = 68	73.7% N = 7,218	93.1% N = 2,708

Table 8 summarizes the percentage of patients who took prescription medications by income group as a ratio to the poverty threshold, USC, and THU status. For patients without a USC, the percentage who took prescription medications was 2.9–3.7 times higher among patients with THU relative to those without. For patients with an income below half the poverty threshold, the percentages who took prescription medications were 23.4% (no USC or THU), 84.8% (THU without USC), 57.6% (USC without THU), and 86.3% (USC and THU).

**Table 8.** Percentage of Patients by Income Group as a Ratio to the Poverty Threshold Who Took a Prescription Medication in the Past 12 Months Based on USC Status and THU

Ratio of Income to the Poverty Threshold	USC: No THU: No	USC: No THU: Yes	USC: Yes THU: No	USC: Yes THU: Yes
**3+**	27.0% N = 2,314	75.5% N = 559	65.5% N = 22,717	88.2% N = 14,392
**2–2.99**	24.2% N = 922	69.9% N = 156	65.7% N = 6,443	89.6% N = 3,567
**1–1.99**	24.8% N = 1,091	75.5% N = 139	66.9% N = 6989	90.1% N = 3,688
**0.5–0.99**	22.2% N = 469	82.5% N = 63	66.9% N = 2,753	91.5% N = 1,593
**< 0.5**	23.4% N = 248	84.8% N = 33	57.6% N = 942	86.3% N = 503

Table 9 shows the modeling estimates of a multivariable logistic regression of taking a prescription medication within the past 12 months for USC, THU, and their interaction. This model adjusted for the measured variables (demographics, chronic conditions, behavioral health conditions, challenges of ability, education, and income group). The estimated association of USC (OR: 3.12, 95% CI: 2.89–3.37, P < 0.001) shows a 3-fold increase in the odds of prescription medication utilization relative to no USC. THU shows a 7-fold increase (OR: 7.39, 95% CI: 6.19–8.84, P < 0.001) in the odds of prescription medication utilization. The interaction of USC and THU (OR: 0.50, 95% CI: 0.41–0.60, P < 0.001) demonstrates that the combination of the factors would show an increase of roughly half of the product of the two odds ratios, which is an approximately 11.5-fold increase in the odds of prescription medication utilization relative to patients without a USC or THU.

**Table 9.** Modeling Estimates for a Multivariable Logistic Regression of Taking a Prescription within the Past 12 Months

Variable	Estimated Odds Ratio	95% CI for Odds Ratio	P-value
**USC**	3.12	(2.89–3.37)	< 0.001
**THU**	7.39	(6.19–8.84)	< 0.001
**Interaction: USC and THU**	0.50	(0.41–0.60)	< 0.001

*Note*. The model estimates the associated odds ratio of categories USC, THU, and their interactions while adjusting for demographics, survey year, chronic conditions, behavioral health conditions, challenges of ability, education, income group, and food barriers.

Table 10 provides a statistical comparison of patients with and without THU in the subset of patients without a USC. Among the demographic variables, patients with THU had statistically significant differences (P <= 0.001) in age, gender, region, and geography. For age, patients with THU had higher percentages in the categories of 18–34, 65–74, and at least 75. For gender, patients with THU had much lower percentages of male patients (47.3%) relative to those without THU (63.9%), which was statistically significant (P < 0.001). For race and ethnicity, patients with THU had a higher percentage of patients who were White or Asian, while patients who were Black, Hispanic, or American Indian/Alaskan Native (AIAN) had lower percentages of THU. For geographic variables, patients with THU had a higher percentage of patients living in the Northeast and West, with lower percentages in the Midwest and South. Patients with THU were less likely to live in rural geographic areas. Patients with THU had significantly higher levels of education, with a larger percentage of college degrees and graduate degrees.

Patients with THU had higher and statistically significantly greater percentages of each of the chronic conditions of diabetes, hypertension, asthma, COPD, cancer, and CHD. Patients with THU also included patients with anxiety and depression at much greater rates. Likewise, for challenges of ability, patients with THU had statistically significantly higher percentages of patients with visual impairments, hearing impairments, or difficulties with raising their arms, self-care, hands or fingers, or errands. While the percentage of patients with communication difficulties was slightly higher among patients with THU, this was not statistically significant. For income groups, patients with THU had a higher percentage with incomes above 3 times the poverty threshold and lower percentages in all other categories, which collectively showed a statistically significant difference in distribution. Food barriers were approximately equally represented among patients with and without THU, with no statistically significant differences in their percentages.

**Table 10:** Statistical Comparisons of Characteristics of Patients with and without THU Among Those with No USC

Variable	Category	No THU	THU	P-value
**Sample Size**		5,044	950	
**Age**	18–34	2,157 (42.8%)	461 (48.5%)	0.001
	35–49	1,493 (29.6%)	250 (26.3%)	
	50–64	950 (18.8%)	139 (14.6%)	
	65–74	312 (6.2%)	72 (7.6%)	
	75+	132 (2.6%)	28 (2.9%)	
**Gender**	Male	3,224 (63.9%)	449 (47.3%)	< 0.001
**Race/Ethnicity**	White	2,870 (56.9%)	636 (66.9%)	< 0.001
	Black	512 (10.2%)	75 (7.9%)	
	Hispanic	1,098 (21.8%)	121 (12.7%)	
	Asian	341 (6.8%)	82 (8.6%)	
	American Indian/Alaska Native (AIAN)	104 (2.1%)	16 (1.7%)	
	Other/Unknown	119 (2.4%)	20 (2.1%)	
**Region (USA)**	Northeast	520 (10.3%)	123 (12.9%)	< 0.001
	Midwest	1,098 (21.8%)	172 (18.1%)	
	South	2,128 (42.2%)	361 (38.0%)	
	West	1,297 (25.7%)	294 (30.9%)	
**Geography**	Rural	641 (12.7%)	68 (7.2%)	< 0.001
**Education**	No College	1,635 (32.4%)	148 (15.6%)	< 0.001
	Some College	750 (14.9%)	122 (12.8%)	
	College Degree	1,935 (38.4%)	437 (46.0%)	
	Graduate Degree	724 (14.4%)	243 (25.6%)	
**Diabetes**		88 (1.7%)	47 (4.9%)	< 0.001
**Hypertension**		638 (12.6%)	198 (20.8%)	< 0.001
**Asthma**		543 (10.8%)	191 (20.1%)	< 0.001
**COPD**		96 (1.9%)	35 (3.7%)	0.001
**Cancer**		162 (3.2%)	63 (6.6%)	< 0.001
**CHD**		61 (1.2%)	23 (2.4%)	0.006
**Anxiety**		424 (8.4%)	298 (31.4%)	< 0.001
**Depression**		500 (9.9%)	314 (33.1%)	< 0.001
**Visual Impairment**		766 (15.2%)	189 (19.9%)	< 0.001
**Hearing Impairment**		509 (10.1%)	126 (13.3%)	0.004
**Difficulty with Communication**		205 (4.1%)	48 (5.1%)	0.193
**Difficulty with Raising Arms**		69 (1.4%)	36 (3.8%)	< 0.001
**Difficulty with Self Care**		71 (1.4%)	45 (4.7%)	< 0.001
**Difficulty with Hands or Fingers**		161 (3.2%)	58 (6.1%)	< 0.001
**Difficulty with Errands**		171 (3.4%)	80 (8.4%)	< 0.001
**Income Ratio to Poverty Threshold**	3 or greater	2,314 (45.9%)	559 (58.8%)	< 0.001
	2–2.99	922 (18.3%)	156 (16.4%)	
	1–1.199	1,091 (21.6%)	139 (14.6%)	
	0.5–0.99	469 (9.3%)	63 (6.6%)	
	Below 0.5	248 (4.9%)	33 (3.5%)	
**Food Barrier**		486 (9.6%)	91 (9.6%)	1.000

## Discussion

Due to its large, nationally representative samples over a period of years, the NHIS data provide information on 5,994 patients without a USC in the period from 2020 to 2022. The study estimates that approximately 8.6% of patients in the United States lack a USC. Extrapolated to the population, this would amount to more than 28 million people in the United States. While some of these patients are quite young and healthy, many others would benefit from more consistent care while managing chronic conditions, behavioral health conditions, or challenges of ability. For the purpose of filling prescriptions, the study's results suggest that telehealth can effectively serve as a substitute for a USC. In the study, the percentage of patients with THU who took prescription medications was on par with patients who have a USC without THU. These results consistently showed a large multiplicative association across a wide range of demographic factors, income groups, chronic conditions, behavioral health conditions, and challenges of ability.

Among patients without a USC, the study shows that the subgroup with THU was substantially different from those without THU in terms of most demographic characteristics, health conditions, behavioral health conditions, challenges of ability, and income group. The patients with THU were in demographic groups with higher rates of overall utilization, had greater percentages of all chronic conditions, anxiety, depression, and nearly every challenge of ability. This does suggest that patients with greater needs are more likely to utilize telehealth. Nevertheless, for patients without a USC, the percentage with THU (15.8%) was less than half of the percentage with THU among those with a USC (37.3%). Taking steps to provide greater technological access and promote the utilization of telehealth would help more patients without a USC to obtain needed prescriptions and ensure more regular healthcare.

Telehealth has the potential to help patients overcome barriers to healthcare access. For patients who live far from healthcare facilities (Ferrer-Roca et al., 2010), who have transportation barriers (Westby et al., 2021), or who have mobility concerns (Forducey et al., 2012), a remote appointment can help patients who otherwise might face considerable challenges in attending in-person appointments. Filling prescriptions for routine medications or for acute illnesses by telehealth is both convenient and can be a direct outcome of the remote appointment (Fuentes et al., 2022). Nevertheless, access to telehealth can involve a wide range of barriers, such as a lack of awareness (Kruse et al., 2016), technological access (Reges et al., 2022), conceptual comfort (Smith & Raskin, 2021), technological comfort (Reges et al., 2022), or materials in the patient's preferred language (Dixit et al., 2021).

The study's results also demonstrate the benefit of telehealth for patients with low income. Overall and among patients with an income below one half the poverty threshold, THU was associated with markedly higher percentages for taking prescription medications. This may suggest that healthcare facilities could better address the needs of patients with low income and promote telehealth as a strategy for obtaining prescriptions as needed.

Further research would help to build the evidence base for the efficacy of telehealth in primary care settings. Electronic health records could be studied to estimate how frequently telehealth is utilized to gain access to or refill prescription medications. Outreach programs for patients in underserved populations could be used to facilitate appointment scheduling and telehealth navigation. Patient surveys could investigate the reasons why patients might prefer telehealth, in-person care, or a combination of the two.

The study includes a few limitations. The data were self-reported, gathered through an interview, and collected at a single time point for each subject. Telehealth and taking prescription medications were only measured with binary variables. Telehealth was not separated by phone or video conference, and it was not measured in terms of its frequency of utilization. The data were gathered during different periods of the COVID-19 pandemic, which influenced the availability of in-person primary care appointments and overall healthcare needs. For prescription medications, not every patient may have needed a prescription, and the gathered data did not measure which medicines were taken or the degree of adherence. The individual telehealth visits may or may not have led to a prescription. Comparisons of telehealth and a usual source of care are comparisons of direct utilization of remote appointments to the availability of in-person appointments. Some patients with a USC may have not had an appointment at all. For these reasons, some caution is advised in interpreting the results. Despite this, the evidence does point to a clear benefit of utilizing telehealth to facilitate access to prescription medications.

## Declarations

Data Availability Statement: The data for this study are publicly available from the Centers for Disease Control's NHIS study website:

https://ftp.cdc.gov/pub/Health_Statistics/NCHS/Datasets/NHIS/2020/adult20csv.zip

https://ftp.cdc.gov/pub/Health_Statistics/NCHS/Datasets/NHIS/2021/adult21csv.zip

https://ftp.cdc.gov/pub/Health_Statistics/NCHS/Datasets/NHIS/2022/adult22csv.zip
